# Beneficial Effects of Repeated Washed Microbiota Transplantation in Children With Autism

**DOI:** 10.3389/fped.2022.928785

**Published:** 2022-06-16

**Authors:** Zhao-Yu Pan, Hao-Jie Zhong, Dong-Ni Huang, Li-Hao Wu, Xing-Xiang He

**Affiliations:** ^1^Department of Gastroenterology, The First Affiliated Hospital of Guangdong Pharmaceutical University, Guangzhou, China; ^2^Research Center for Engineering Techniques of Microbiota-Targeted Therapies of Guangdong Province, Guangzhou, China; ^3^School of Biology and Biological Engineering, South China University of Technology, Guangzhou, China

**Keywords:** autism, clinical efficacy, gastrointestinal symptoms, sleep disorders, treatment course, washed microbiota transplantation

## Abstract

**Objective:**

While fecal microbiota transplantation is demonstrated to improve symptoms of autism spectrum disorder (ASD), it remains unclear whether additional treatment courses yield better results. This study sought to evaluate the efficacy of repeated washed microbiota transplantation (WMT) in children with ASD.

**Methods:**

Retrospective data from children who were serially treated with WMT, including ASD symptoms, sleep disorders, gastrointestinal (GI) symptoms, and white blood cell (WBC) and globulin levels were obtained. The effect of WMT on children with ASD and whether additional WMT courses led to a further improvement in symptoms were assessed.

**Results:**

Aberrant Behavior Checklist (ABC), Childhood Autism Rating Scale, and Sleep Disturbance Scale for Children (SDSC) scores, the proportion of children with constipation and abnormal fecal forms, and WBC and globulin levels were all significantly lower in ASD children after WMT. More WMT treatment courses led to significantly lower scores on the ABC and SDSC.

**Conclusion:**

WMT significantly improved ASD and GI symptoms and sleep disorders in children with ASD, and reduced systemic inflammation. Additional WMT courses led to more obvious improvements in ASD symptoms within three treatment courses.

## Introduction

Autism spectrum disorder (ASD) manifests in the first 3 years of life (1) and is primarily characterized by impaired social functioning, repetitive behavior, and restricted communication (2). The global prevalence of ASD was about 1% in 2016 (3). While the prevalence of ASD is increasing, there are still no effective treatments.

The gut microbiota plays a critical role in ASD and differs significantly between individuals with this disorder and those without (4). Studies indicate that the gut microbiota is associated with ASD-like phenotypes in murine models (5) and some interventions that target the gut microbiota, such as treatment with the probiotic Lactobacillus reuteri (6), are able to reverse social deficits in ASD mice. The effect of probiotics on autistic behavior in children is general and does not differ significantly from its effect on healthy children (7); however, it remains unknown whether this is due to low probiotic engraftment in the gut (8).

Fecal microbiota transplantation (FMT) is the delivery of a fecal suspension from a healthy donor to a patient’s gastrointestinal (GI) tract to reconstruct the normal gut microbiota and alleviate conditions such as Clostridiodes difficile infection, ulcerative colitis, and various neurological diseases (9, 10). ASD mice receiving gut microbiota from healthy mice showed reduced autism-like behaviors (11). To date, two cohort studies have shown that FMT can improve ASD symptoms in humans (12, 13), but it remains unclear whether more treatment courses yield greater improvement. In addition, FMT use is limited by the high incidence of adverse events (AEs) such as fever, abdominal pain, and abdominal bloating, as well as the complexity of manually preparing FMT samples (14).

Washed microbiota transplantation (WMT) can significantly reduce FMT-related AEs by removing parasite eggs, fecal particles, and fungi through a series of automated washing procedures (15). The current study sought to evaluate the effect of WMT on ASD symptoms, sleep disorders, GI symptoms, and systemic inflammation, and to determine the optimal number of WMT courses for children with ASD.

## Materials and Methods

### Study Design and Participants

This was a retrospective study conducted at the First Affiliated Hospital of Guangdong Pharmaceutical University. Approval was obtained (#2020-14) from the Ethics Committee of the First Affiliated Hospital of Guangdong Pharmaceutical University, and the protocol was in accordance with the Helsinki Declaration. Informed consent was provided by each child’s parents.

ASD children who had received at least two consecutive WMT courses at our hospital from 1 June 2019 to 30 June 2021 were included in the study. ASD was diagnosed based on criteria from the 5th edition of the Diagnostic and Statistical Manual of Mental Disorders. Children were excluded if they (i) used antibiotics or probiotics within 1 month before WMT, (ii) were diagnosed with ulcerative colitis, Crohn’s disease, celiac disease, major brain malformations, or other serious heart, lung, liver, or kidney disease, or (iii) required emergency treatment for serious GI or other life-threatening problems.

### Washed Microbiota Preparation and Transplantation

Stools from healthy donors were screened for WMT as previously described (16). In brief, healthy donors were asked to fill out a questionnaire that included the Hamilton Depression and Hamilton Anxiety Scales to exclude individuals with any risk factors. Doners also underwent physical examinations, blood and stool tests, and other laboratory screenings to exclude those with communicable diseases. The 15 anonymous donors did not live in the same households, and their fecal microbiota were assessed using standardized management processes (17). Fecal suspensions from the various donors were randomly assigned to each child. Washed microbiota suspensions were prepared using an automatic machine (GenFMTer; FMT Medical, Nanjing, China) as previously described (15). In brief, feces were suspended in normal saline (100 g per 500 mL) and filtered with a GenFMTer. The fecal suspension was then centrifuged at 700 × *g* for 3 min, and the supernatant was discarded. The microbiota pellet was washed with normal saline and centrifuged three times. The final sediment obtained from 100 g of feces was suspended with 100 mL of normal saline.

**FIGURE 1:**
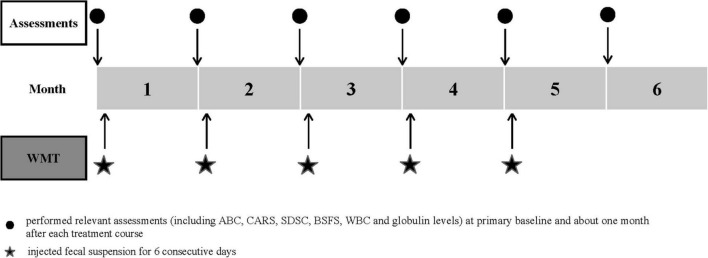
Study design timeline. The time schedule of relevant assessments and treatment courses. ABC, Aberrant Behavior Checklist; BSFS, Bristol Stool Form Scale; CARS, Childhood Autism Rating Scale; SDSC, Sleep Disturbance Scale for Children; WBC, white blood cell; WMT, washed microbiota transplantation.

For WMT, the fecal suspension (about 5.0 × 10^13^ bacteria) was injected (60–90 mL per day for 6 consecutive days) through a transendoscopic enteral tube. According to the Nanjing consensus on WMT methodology, only fresh healthy microbiota were used. The children were asked to stay in a right lateral position for at least 2 h after transplantation and recommended to eat light meals for at least 2 days after WMT. The time interval between each treatment course was 1 month (Figure 1).

### Data Collection and Definitions

Demographic data, body mass index (BMI), ASD symptoms, sleep disorders, GI symptoms, therapies for ASD symptoms (including behavioral and communication therapy, educational therapy, or medication), and white blood cell (WBC) and globulin levels were extracted from electronic and paper medical records.

ASD symptoms were assessed using the Aberrant Behavior Checklist (ABC) and Childhood Autism Rating Scale (CARS). The ABC was filled out by each child’s parents and assessed five common areas, stereotypy, irritability, lethargy, inappropriate speech, and hyperactivity. A score > 53 points indicated that there was a high possibility of ASD (13, 18). The CARS is a 15-item scale that can be used to confirm ASD and further evaluate symptom severity (30–36, light to moderate; > 36, severe) (19). This scale was completed by a pediatrician who was blinded to the treatment. The Sleep Disturbance Scale for Children (SDSC) was completed by the parents and a score > 39 was suggestive of a sleep disorder (20). GI symptoms were evaluated by stool consistency and the occurrence of constipation. Stool consistency was assessed using the Bristol Stool Form Scale (BSFS) (1 = very hard, 7 = liquid, 3–5 = normal fecal form) (21), while constipation was defined using the Rome III criteria (22). Relevant assessments were performed at primary baseline and approximately 1 month after each treatment course.

### Statistical Analyses

Statistical analyses were performed using SPSS 23.0 (IBM Corp., Armonk, NY, United States) and Prism 8 software (GraphPad Prism Inc., San Diego, CA, United States). Continuous data are presented as the mean and standard deviation for normally distributed data or median (quartile range) for non-normally distributed data. Categorical variables are given as frequencies and percentages. Independent categorical variables were analyzed using Fisher’s exact or Chi-square tests. Comparisons were conducted using McNemar’s tests for paired categorical variables. The Pearson and Spearman correlation tests were used for normally and non-normally distributed data, respectively. Significant differences between two independent groups were evaluated using unpaired Student’s *t*-tests (normally distributed) or Mann–Whitney *U*-tests (non-normally distributed). Differences between paired data were evaluated using paired Student’s *t*-tests (normally distributed) or Wilcoxon signed-rank tests (non-normally distributed). Differences were considered significant at *P* < 0.05.

## Results

### Clinical Characteristics of Children Who Underwent Washed Microbiota Transplantation

A total of 55 children received inclusion and exclusion criteria screening, of whom 42 (34 males and 8 females) were enrolled in the study. Thirty children completed two WMT courses, 23 children completed three courses, 14 children completed four courses, and 6 children completed five courses. Of the enrollees, 29 received both WMT and other therapies (including behavioral and communication therapy, educational therapy, or medication) and 13 received WMT alone. Participants had a median age of 6 years and 80.95% (34/42) were male. The median disease duration was 3.26 (1.15–6.01) years, and 83% of children with ASD had a sleep disorder at primary baseline. Other demographic and clinical characteristics are outlined in Table 1.

**TABLE 1:** Baseline characteristics of patients.

Clinical characteristic	Patients with ASD (*n* = 42)
Age (years)	6.00 (3.75–8.25)
Male (%)	34 (80.95%)
BMI (kg/m^2^)	17.01 (15.62–18.60) (*n* = 40)
Disease duration (years)	3.26 (1.15–6.01) (*n* = 42)
ABC	59.00 (49.75–72.00)
CARS	36.00 ± 3.77 (*n* = 41)
SDSC	52.50 ± 12.78
BSFS	3.00 (1.00–4.00) (*n* = 40)
Constipation (%)	21 (50%)

*Data are presented as the mean ± standard deviation, median (interquartile range), or n (%). ABC, Aberrant Behavior Checklist; ASD, autism spectrum disorder; BMI, body mass index; BSFS, Bristol Stool Form Scale; CARS, Childhood Autism Rating Scale; SDSC, Sleep Disturbance Scale for Children.*

### Effect of Washed Microbiota Transplantation on Autism Spectrum Disorder Symptoms and Sleep Disorders

**FIGURE 2:**
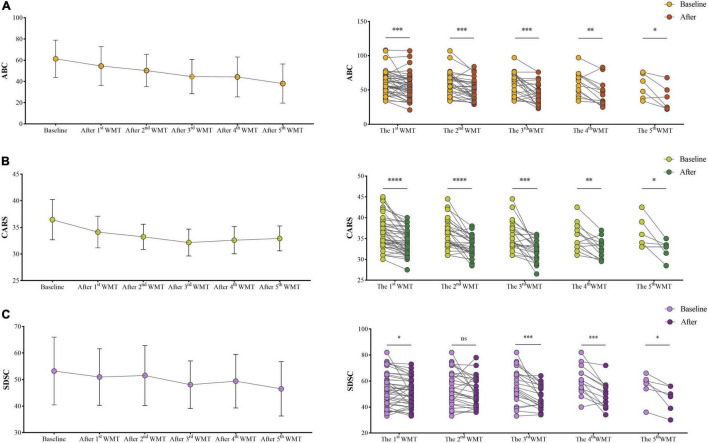
Effect of WMT on ASD symptoms and sleep disorders. Changes in ABC **(A)**, CARS **(B)**, and SDSC **(C)** scores. The line chart shows the means and standard deviation of the data. Paired data from each course are compared with their primary baseline. ABC, Aberrant Behavior Checklist; CARS, Childhood Autism Rating Scale; SDSC, Sleep Disturbance Scale for Children; WMT, washed microbiota transplantation. Statistical analysis was performed using paired Student’s *t*-tests or Wilcoxon signed-rank tests. *P < 0.05; ^**^P < 0.01; ^***^P < 0.001; ^****^P < 0.0001; ^ns^*P* > 0.05 not significant.

The effect of WMT on ASD symptoms was determined using three commonly used scales. ABC, CARS, and SDSC scores all decreased as the number of WMT courses increased (Figure 2). The ABC (Figure 2A), CARS (Figure 2B), and SDSC scores of ASD children were significantly lower following WMT than at primary baseline, although the difference in SDSC scores did not remain significant after the second WMT (Figure 2C).

### Effect of Washed Microbiota Transplantation on Gastrointestinal Symptoms

**FIGURE 3:**
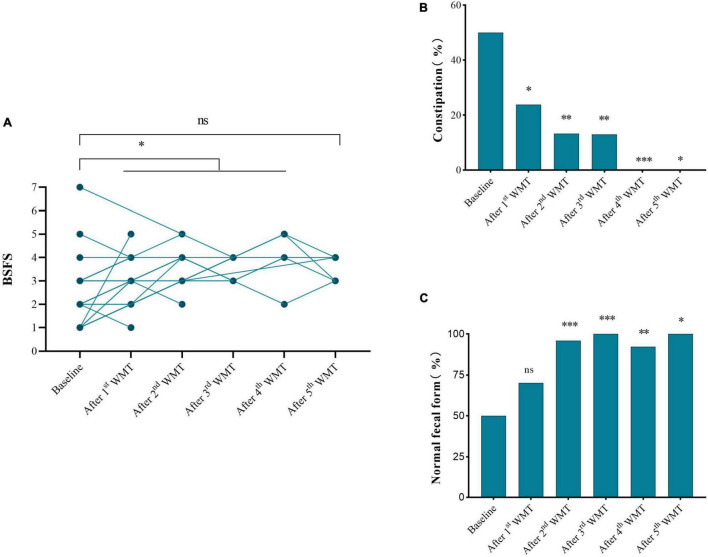
Effect of WMT on gastrointestinal symptoms. **(A)** Changes in BSFS score. **(B)** Changes in the proportion of normal fecal form. **(C)** Changes in the proportion of subjects with constipation. Data from each course compared with their primary baseline. BSFS, Bristol Stool Form Scale; WMT, washed microbiota transplantation. Significance was determined using Wilcoxon signed-rank, Fisher’s exact, and Chi-square tests. *P < 0.05; ^**^P < 0.01; ^***^P < 0.001; *^ns^P* > 0.05 not significant.

Half (21/42) of children had abnormal fecal form and constipation at primary baseline (Figures 3B,C). After WMT, the BSFS scores of children were 3–5 (Figure 3A) and the proportion with normal fecal form gradually increased with additional WMT courses (Figure 3B). Moreover, the proportion of children with constipation gradually declined with additional WMT courses (reduced to zero after the fourth WMT) (Figure 3C).

### Effect of Washed Microbiota Transplantation on Systemic Inflammation

**FIGURE 4:**
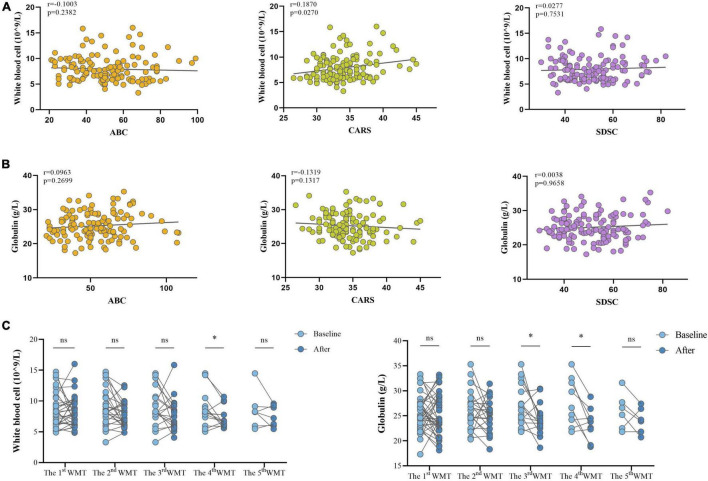
Effect of WMT on WBC and globulin levels in children with ASD. **(A)** Spearman correlations between WBC counts and clinical scores (ABC, CARS, and SDSC). **(B)** Spearman correlations between globulin levels and clinical scores (ABC, CARS, and SDSC). **(C)** WBC and globulin levels before and after WMT. Paired data are from each course compared with their primary baseline. ABC, Aberrant Behavior Checklist; CARS, Childhood Autism Rating Scale; SDSC, Sleep Disturbance Scale for Children; WBC, white blood cell; WMT, washed microbiota transplantation. Spearman correlation and Wilcoxon signed-rank tests were used to determine statistical significance. *P < 0.05, ^ns^*P* > 0.05 not significant.

It was next determined whether systemic inflammation was associated with ASD and whether WMT had an impact on inflammatory markers. WBC counts were positively correlated with CARS score (*r* = 0.1870, *P* = 0.0270; Figure 4A), and were significantly lower after the fourth WMT than at primary baseline (Figure 4C). Globulin levels were significantly reduced after the third and fourth WMT but were not significantly correlated with ABC, CARS, or SDSC scores (Figures 4B,C).

### Effect of an Increasing Number of Washed Microbiota Transplantation Courses on Autism Spectrum Disorder Assessments

**FIGURE 5:**
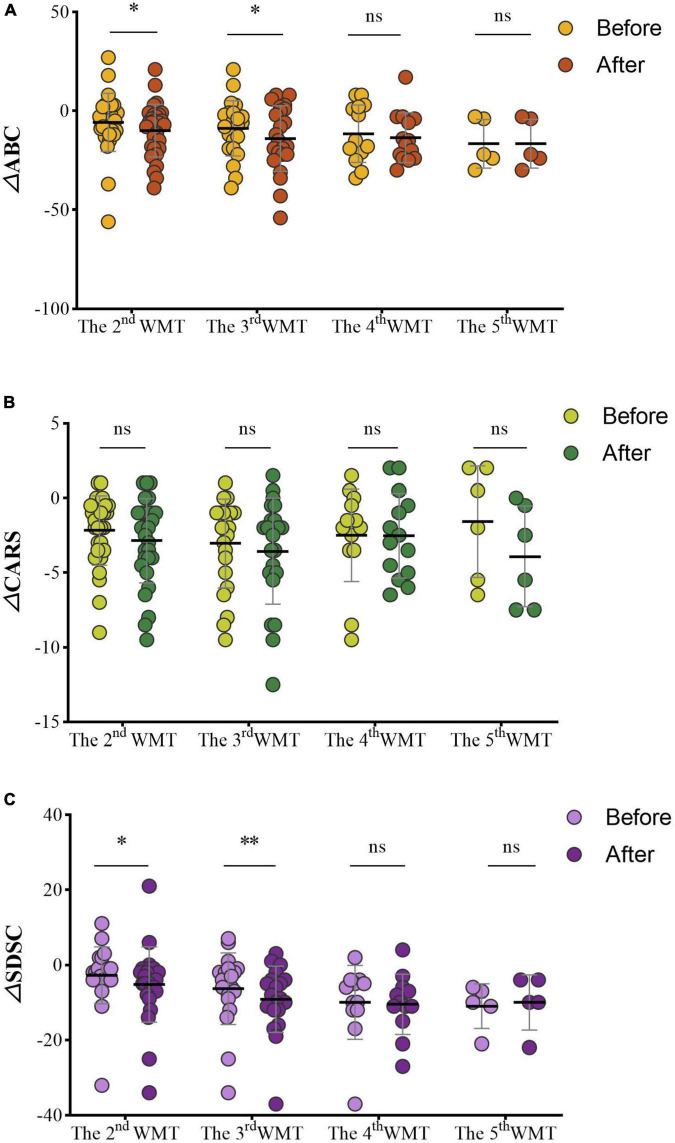
Effect of different numbers of WMT courses on the ABC **(A)**, CARS **(B)**, and SDSC **(C)**. △ABC: the ABC after WMT minus ABC at primary baseline; △CARS: the CARS after WMT minus CARS at primary baseline; △SDSC: the SDSC after WMT minus SDSC at primary baseline. ABC, Aberrant Behavior Checklist; CARS, Childhood Autism Rating Scale; SDSC, Sleep Disturbance Scale for Children; WMT, washed microbiota transplantation. Paired Student’s *t*-tests and Wilcoxon signed-rank tests were used to compare the data. *P < 0.05; ^**^P < 0.01; ^ns^*P* > 0.05 not significant.

Changes of the ASD scales before and after each additional WMT course (△ABC, △CARS, △SDSC) were assessed. Within WMT sessions, more courses led to significantly lower ABC scores [△ABC: second WMT vs. first WMT: −6.50 (−19.00, −2.00) vs. −5.00 (−10.50, 2.25), *P* = 0.045; third WMT vs. second WMT: −14.04 ± 16.62 vs. −8.83 ± 13.96, *P* = 0.022; Figure 5A] and SDSC scores [△SDSC: second WMT vs. first WMT: −4.50 (−7.75, −1.00) vs. −2.00 (−4.00, −1.00), *P* = 0.012; third WMT vs. second WMT: −8.50 (−12.00, −4.00) vs. −4.50 (−8.75, −1.25), *P* = 0.0045; Figure 5C]. CARS scores gradually decreased with additional WMT courses, but there were no statistically significant differences between two adjacent courses (Figure 5B).

Scores on the ABC (△ABC: fourth WMT vs. third WMT: −13.57 ± 12.56 vs. −11.57 ± 14.48, *P* = 0.527; fifth WMT vs. fourth WMT: −19.40 ± 15.32 vs. −16.60 ± 12.32, *P* = 0.351; Figure 5A), CARS [△CARS: fourth WMT vs. third WMT: −2.75 (−5.13, −0.25) vs. −2.00 (−3.50, −0.38), *P* = 0.930; fifth WMT vs. fourth WMT: −3.92 ± 3.38 vs. −1.58 ± 3.73, *P* = 0.084; Figure 5B] and SDSC [△SDSC: fourth WMT vs. third WMT: −10.50 (−14.00, −6.25) vs. −8.00 (−12.00, −4.25), *P* = 0.608; fifth WMT vs. fourth WMT: −10.00 ± 7.35 vs. −11.00 ± 5.96, *P* = 0.840; Figure 5C] decreased in the fourth and fifth courses compared to previous courses, but the difference was not statistically significant.

### Effect of an Increasing Number of Washed Microbiota Transplantation Courses on Gastrointestinal Symptoms

Changes in GI symptoms (including constipation and normal fecal form) before and after each additional WMT course were measured. The proportion of ASD children with constipation was significantly reduced from 50% at primary baseline to 23.81% after the first WMT (*P* = 0.001, Table 2). The proportion of ASD children with normal fecal form was obviously increased from 48.65% at primary baseline to 70.27% after the first WMT (*P* = 0.008).

**TABLE 2:** Effect of different numbers of WMT courses on the gastrointestinal symptoms of ASD children.

	Baseline	After 1st WMT	After 1st WMT	After 2nd WMT	After 2nd WMT	After 3rd WMT	After 3rd WMT	After 4th WMT	After 4th WMT	After 5th WMT
Constipation (n)	21 (50.00%) (*n* = 42)	10 (23.81%) (*n* = 42)	7 (23.33%) (*n* = 30)	4 (13.33%) (*n* = 30)	4 (17.39%) (*n* = 23)	3 (13.04%) (*n* = 23)	1 (6.25%) (*n* = 15)	0 (0) (*n* = 15)	0 (0) (*n* = 7)	0 (0) (*n* = 7)
*P*	**0.001**		0.375		1.000		1.000		−	
Normal fecal form (n)	19 (51.35%) (*n* = 37)	26 (70.27%) (*n* = 37)	18 (75.00%) (*n* = 24)	23 (95.83%) (*n* = 24)	17 (100%) (*n* = 17)	17 (100%) (*n* = 17)	11 (100%) (*n* = 11)	10 (90.91%) (*n* = 11)	5 (83.33%) (*n* = 6)	6 (100%) (*n* = 6)
*P*	**0.016**		0.125		−		1.000		1.000	

*Data are presented as n (%). WMT, washed microbiota transplantation. P-values based on McNemar’s tests. Significant P-values are in bold.*

### Effect of an Increasing Number of Washed Microbiota Transplantation Courses on Systemic Inflammation

**FIGURE 6:**
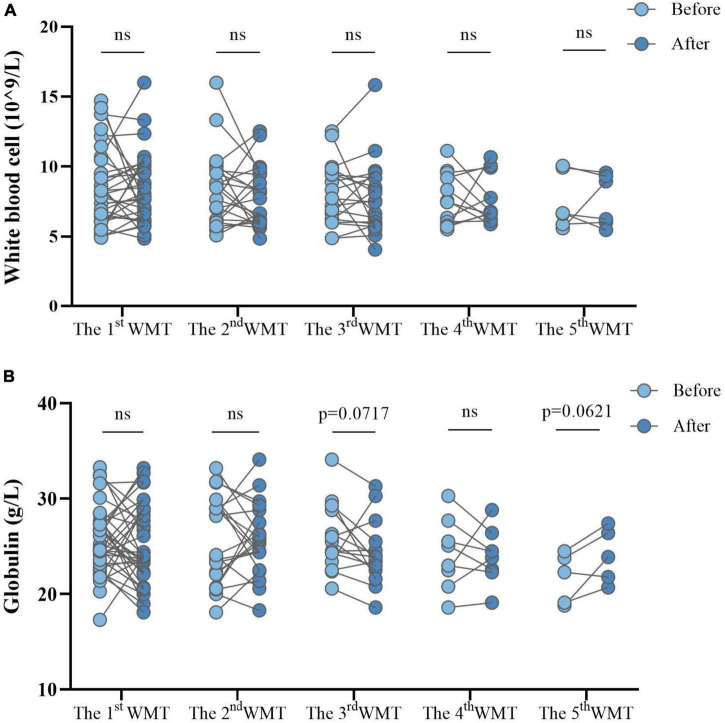
Effect of different numbers of WMT courses on systemic inflammation measured by white blood cell counts **(A)** and globulin levels **(B)**. WMT, washed microbiota transplantation. Paired Student’s *t*-tests and Wilcoxon signed-rank tests were used to compare the data. ns, *P* > 0.05 not significant.

Changes in systemic inflammation before and after each additional WMT course in ASD children were evaluated. WBC counts (Figure 6A) and globulin levels (Figure 6B) remained similar before and after each course.

## Discussion

Few clinical studies have examined the effect of FMT on ASD. Li et al. (13) found that the symptoms of children with ASD improved significantly after FMT treatment, but improvements were reversed after treatment was stopped for 4 weeks. The WMT protocol and methods used in this study to measure symptoms were unclear. The current study assessed ASD-related symptoms, sleep disorders, and GI symptoms after WMT. In individuals who received three treatment courses, additional courses led to more obvious improvements in ASD-related symptoms and sleep disorders.

Alterations in the gut microbiota composition of children with ASD are associated with both GI and nervous system-specific symptoms (23, 24). Several studies have reported that children with ASD have lower gut microbiota diversity and richness than healthy controls (25, 26) as well as lower abundances of beneficial bacteria [such as Bifidobacterium (27, 28) and Prevotella (29)]. WMT is shown to attenuate ASD symptoms by altering the gut microbiota (12, 13). The current study also found that the ABC and CARS scores were significantly lower after WMT. In addition, Kang et al. (12) showed that the bacterial diversity and relative abundances of Bifidobacterium and Prevotella increased significantly after FMT in ASD children, and the unweighted UniFrac distance between the host gut and donor sample decreased significantly after WMT. Li et al. (13) also demonstrated that the ASD microbiome more closely resembled that of healthy individuals and donors after FMT. These findings indicate that FMT can cause the gut microbiota of ASD children to more closely resemble the microbiota of donors and increase the abundance of beneficial bacteria.

The microbiome–gut–brain axis is implicated in ASD pathogenesis (30, 31) and the following mechanisms may help to explain how WMT relieves ASD symptoms. First, FMT may improve ASD-like behavior by decreasing neurotransmitter levels. For example, serum 5-hydroxytryptamine (5-HT) levels are significantly higher in ASD children than controls (32). An open-label study found that FMT may decrease the levels of 5-HT, a monoamine neurotransmitter that can modulate mood, behavior, and neurodevelopment (13). Second, some studies have shown that fecal metabolites, such as p-cresol and gamma-aminobutyric acid (33, 34), which can penetrate the blood–brain barrier and affect neural function (35), are altered in ASD children. After FMT, p-cresol sulfate levels in children with ASD are similar to those of neurotypical children (36), suggesting that this procedure can also reduce fecal metabolite levels. Finally, WMT may indirectly improve ASD symptoms by decreasing inflammatory cytokine levels. Several pro-inflammatory cytokines, such as interleukin (IL)-6 and tumor necrosis factor-α, are significantly higher in the peripheral blood of ASD patients than in healthy controls (37). FMT is also associated with lower serum IL-6 levels in patients with ulcerative colitis, indicating that this procedure may alleviate inflammatory diseases by reducing inflammatory cytokine production (38).

A recent meta-analysis indicated that ASD children had lower sleep quality than healthy controls (39), and that sleep disorders may aggravate ASD symptoms (40, 41). About 83% of the children in the current study had sleep disorders and WMT improved their SDSC scores. Lower gut microbiome diversity and richness and higher IL-1β levels are associated with insomnia (42). FMT was shown to decrease IL-1β production in a mouse model of colitis (43). A clinical study found that FMT could increase microbiota diversity and improve sleep (44). Thus, WMT may affect sleep by increasing microbiota diversity and decreasing inflammatory cytokine production.

ASD children have various GI symptoms (45), especially constipation (46), which is partly due to dysbiosis of the gut microbiome, including high levels of Escherichia/Shigella and Clostridium cluster XVIII (30). FMT regulates the gut microbiota to alleviate chronic functional constipation (47). In addition, average Gastrointestinal Symptom Rating Scale scores and the occurrence of no stool and hard stool significantly declined after FMT (12, 13). The current study also showed that WMT led to a decline in the proportion of children with constipation and more normal fecal form, indicating that FMT can improve GI symptoms in ASD children.

Systemic inflammation is also a characteristic of ASD (48). Acute-phase reactant (inflammatory biomarker) levels were elevated in ASD children (49), while serum γ globulin levels, which correlate with inflammation, were decreased (50). Results from the current study showed that WBC levels correlated positively with CARS scores. Moreover, WBC and globulin levels in ASD children decreased after WMT. These findings suggest that WMT improved ASD symptoms by reducing the levels of inflammatory indicators.

While prior studies have reported that FMT improves ASD-related symptoms, it is unclear whether a single treatment can have a long-term effect and whether more treatment courses can have a greater impact on symptom resolution. In the current study, ABC and SDSC scores decreased significantly with additional WMT courses. These findings supported a previous report from our center that described the relationship between the number of WMT courses and its effects on patients with non-erosive reflux disease (51). He et al. also showed that multiple fresh FMTs had a stronger impact on patients with Crohn’s disease than a single fresh FMT (52). Results from the current study indicated that three or more WMT courses led to more obvious improvements in ASD children.

This study has some potential limitations. First, owing to its retrospective nature, the intestinal microbiota and its metabolites were not measured before and after WMT. Thus, the mechanism by which WMT improves ASD symptoms and the responsible microbiota species remain unknown. In addition, C-reactive protein and levels of inflammatory factors such as IL-6, IL-10, and TNF-a, were unavailable to assess before and after WMT. Second, confounders such as dietary changes and other treatments which may affect ASD symptoms, cannot be excluded (53). Third, the sample size was small. Our results should be confirmed in a large, prospective, multi-center study (54).

## Conclusion

WMT can significantly improve ASD and GI symptoms, reduce sleep disorders in children with ASD, and lower systemic inflammation. Additional WMT courses led to more obvious improvements in the symptoms of ASD within three treatment courses. However, these results should be replicated in larger studies.

## Data Availability Statement

The raw data supporting the conclusions of this article will be made available by the authors, without undue reservation.

## Ethics Statement

The studies involving human participants were reviewed and approved by the Ethics Committee of the First Affiliated Hospital of Guangdong Pharmaceutical University. Written informed consent to participate in this study was provided by the participants’ legal guardian/next of kin.

## Author Contributions

X-XH and H-JZ designed and coordinated the study. Z-YP and H-JZ participated in data collection. Z-YP and D-NH performed the data analysis. All authors participated in interpreting the data and drafting the manuscript and have read and agreed to the published version of the manuscript.

## Conflict of Interest

The authors declare that the research was conducted in the absence of any commercial or financial relationships that could be construed as a potential conflict of interest.

## Publisher’s Note

All claims expressed in this article are solely those of the authors and do not necessarily represent those of their affiliated organizations, or those of the publisher, the editors and the reviewers. Any product that may be evaluated in this article, or claim that may be made by its manufacturer, is not guaranteed or endorsed by the publisher.
